# Abstinence from Cocaine-Induced Conditioned Place Preference Produces Discrete Changes in Glutamatergic Synapses onto Deep Layer 5/6 Neurons from Prelimbic and Infralimbic Cortices

**DOI:** 10.1523/ENEURO.0308-17.2017

**Published:** 2017-12-13

**Authors:** José I. Pena-Bravo, Carmela M. Reichel, Antonieta Lavin

**Affiliations:** Department of Neuroscience, Medical University of South Carolina, Charleston, SC 29425

**Keywords:** cocaine, glutamate, memory, neuroadaptations, prefrontal cortex, synaptic

## Abstract

Glutamatergic signaling in the medial prefrontal cortex (mPFC) plays a critical role in drug addiction and relapse. The mPFC is functionally subdivided into dorsal (prelimbic, PL) and ventral (infralimbic, IL) regions, and evidence suggests a differential role of these two divisions in the control of drug seeking and taking; however, there is a dearth of information on the cocaine-induced adaptations in PL- and IL-mPFC synaptic glutamate transmission and their regulation of behavioral responses to cocaine-associated stimuli. We tested male rats in a cocaine-induced conditioned place preference (CPP) paradigm. In vitro whole-cell recordings were performed at different abstinence intervals to investigate the neuroadaptations in synaptic glutamate transmission in PL- and IL-mPFC deep layer (5/6) pyramidal neurons. Our results show that in naïve animals, PL-mPFC neurons expressed higher frequency of spontaneous events (sEPSCs) than IL-mPFC neurons. Following cocaine-CPP and a short abstinence (SA) period (8 d), we observed decreases in the amplitude of sEPSCs in both mPFC regions. Longer abstinence periods (30 d), resulted in a sustained decrease in the frequency of sEPSCs and an increase in AMPA receptor rectification only in PL-mPFC neurons. In addition, PL-mPFC neurons expressed a decrease in the area under the curve of sEPSCs, suggesting altered receptor activation dynamics. Synaptic glutamate transmission was not significantly different between retested and naïve rats. These results suggest that retention of cocaine-CPP requires differential modulation of glutamate transmission between PL- and IL-mPFC neurons and that these adaptations are dependent on the abstinence interval and reexposure to the cocaine context.

## Significance Statement

Addicted individuals have cognitive impairments associated with abnormal prefrontal cortex (PFC) function. Preclinical studies suggest that prelimbic (PL)- and infralimbic (IL)-medial PFC (mPFC) glutamatergic output neurons play opposing roles in the control of addiction-related behaviors. Therefore, we used PFC slice recordings from male rats after abstinence from cocaine-conditioned place preference (CPP) to measure the cocaine-evoked changes in glutamate transmission. We show for the first time that in naïve rats, PL-mPFC neurons exhibit a higher frequency of spontaneous excitatory currents compared to IL-mPFC neurons. In addition, synaptic glutamate transmission is selectively altered in rats that are not exposed to the conditioning context; and prolonged abstinence (PA) from cocaine-CPP produces an overall increase in the kinetics of spontaneous excitatory currents. Reversing these glutamate changes might prevent retention of cocaine-context associations.

## Introduction

Addiction treatment studies have demonstrated the need for novel biomarkers that target the symptoms associated with abstinence from drug use (Sinha, 2011). The prefrontal cortex (PFC) processes information relevant for the regulation of addiction-related behaviors, such as: impulse inhibition, behavioral monitoring, and decision-making; among other behaviors (Mansouri et al., 2009; Goldstein and Volkow, 2011; Kim and Lee, 2011; Coutlee and Huettel, 2012). In addicted individuals, the loss of control over drug taking despite negative consequences is one of the most significant symptoms and suggests cognitive deficits associated with abnormal PFC activity (for a review, see Goldstein and Volkow, 2011). Similarly, preclinical studies in rodents have demonstrated that cognitive dysfunction following prolonged cocaine exposure is mediated by altered PFC activity (Briand et al., 2008; George et al., 2008; Ghasemzadeh et al., 2011).

The role of the glutamatergic projections from medial PFC (mPFC) to the nucleus accumbens (NAc) in mediating cocaine seeking behavior has been extensively studied (McFarland et al., 2003; Stefanik et al., 2013; Gipson et al., 2014; McGlinchey et al., 2016; Stefanik et al., 2016). Indeed, in rats trained to self-administer cocaine followed by extinction of the operant response, activity in the prelimbic mPFC (PL-mPFC) was been shown to drive cocaine-seeking behavior, while the infralimbic mPFC (IL-mPFC) plays an opposite role (McLaughlin and See, 2003; Peters et al., 2008; Van den Oever et al., 2010; LaLumiere et al., 2012). These discrete PFC subregions send glutamatergic projections to specific targets in the ventral striatum. PL-mPFC projections innervate mainly the core of the NAc while IL-mPFC projections target the shell of the NAc (Heidbreder and Groenewegen, 2003; Vertes, 2004).

Whereas much of the research on cocaine addiction has focused on the NAc, less is known about long-term synaptic changes occurring in mPFC pyramidal projection neurons as a consequence of the reinforcing properties of a cocaine-associated context. We hypothesize that neurons within the PL-mPFC will express a strengthening of synaptic transmission that promotes the retention of the cocaine-context associations. In contrast, IL-mPFC neurons will show no change in glutamatergic synaptic markers, suggesting a mPFC subregion-specific enhancement of synaptic glutamate transmission in cocaine-context associations. The following study used whole-cell electrophysiological recordings from deep layer 5/6 pyramidal neurons in rats that underwent cocaine-induced conditioned place preference (CPP), followed by different abstinence intervals, to understand the differences in synaptic glutamate neuroadaptations between PL- and IL-mPFC neurons. These findings will inform the differential impact cocaine has in the mPFC and the particular role each region plays in mediating the reinforcing effects of a cocaine-conditioned context.

## Materials and Methods

### Laboratory animals

Subjects were adult male Sprague–Dawley rats (Harlan), weighing 250–275 g on arrival. Rats were pair-housed in a temperature-controlled colony room on a 12/12 h light/dark cycle with food and water available *ad libitum*. All procedures were conducted in accordance with the Guide for the Care and Use of Laboratory Rats (Institute of Laboratory Animal Resources on Life Sciences, National Research Council) and approved by the Institutional Animal Care and Use Committee of the Medical University of South Carolina.

### Apparatus

A three-compartment chamber (68 × 21 × 21 cm; ENV-013; MED Associates) was used to assess CPP. The chamber had manual sliding guillotine doors to separate the three compartments. The neutral compartment in the middle (12 × 21 × 21 cm) had gray walls and floor. The end compartments had the same dimensions (28 × 21 × 21 cm), with the left compartment having black walls with a stainless-steel grid rod floor and the right compartment having white walls with a stainless-steel mesh floor. A computer controlled the CPP test using Med-IV software. A series of infrared photobeams (six beams in the black and white compartments and three beams in the gray compartment) were used to record the amount of time spent in each compartment.

### Cocaine place preference

**Figure 1.**
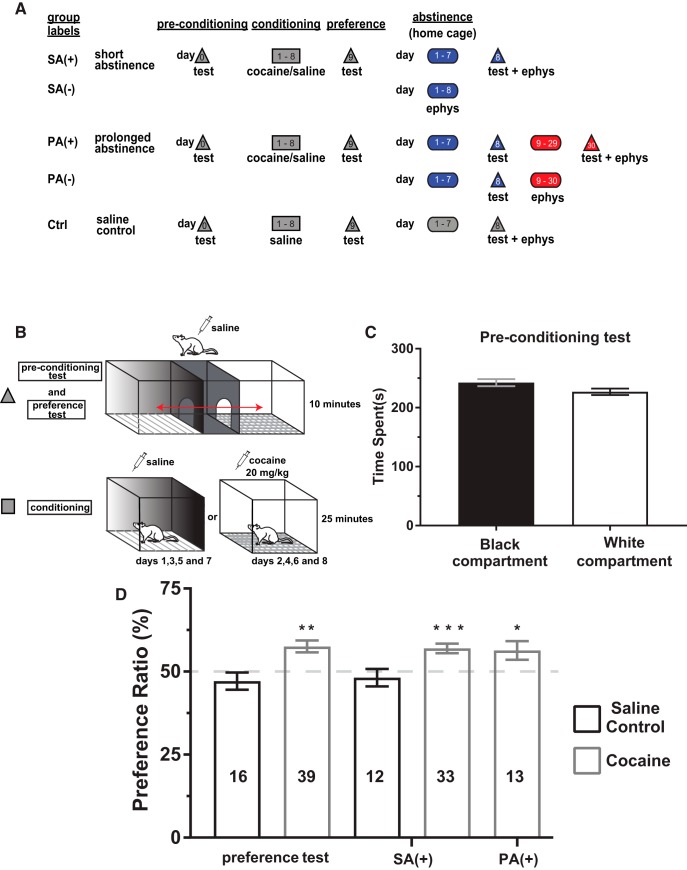
Cocaine-CPP is retained following SA and PA from cocaine experience. ***A***, Timeline of experiments with labels for each experimental group. ***B***, Place preference apparatus diagram with details for each stage of the cocaine-induced place conditioning procedure. ***C***, Time spent in the black and white compartments during the preconditioning (PC) test confirms the unbiased construction of the testing apparatus. ***D***, Preference ratio [(time in cocaine paired compartment/time spent in both compartments) × 100]. Saline-treated (black border) rats and cocaine-conditioned (gray border) rats tested 24 h after the last day of conditioning, after 8 d of abstinence [SA(+)] and after 30 d of abstinence [PA(+)]; *p = 0.0436, **p = 0.0002, ***p < 0.0001 indicate significantly above 50%.

On habituation day (day 0), all rats were allowed to roam the three compartments of the CPP apparatus for 10 min. This habituation day was used as a preconditioning (PC) test to verify the unbiased construction of the apparatus. Conditioning compartments were assigned in an unbiased manner such that each rat had equal opportunity to receive cocaine in their naturally least or most preferred side (Mueller and Stewart, 2000; Tzschentke, 2007; Reichel et al., 2010). Placements were counterbalanced according to chamber color (black/white) and whether the rats received cocaine or saline injections on the first or second day of conditioning. Six cohorts of rats were used in this study and a group of saline-treated (Control rats (Ctrl): cocaine naïve) rats was included in each cohort. Rats were conditioned for 8 d with 24-h intervals between sessions. During conditioning, rats were restricted to either the black or white side for 25 min. During odd days (days 1, 3, 5, and 7) of conditioning, rats placed in their paired compartment (CS+) were injected with cocaine (20 mg/kg) and rats placed in their unpaired compartment (CS-) received a saline injection immediately before compartment placement. During even days (days 2, 4, 6, and 8) of conditioning, treatments were alternated, and rats were placed in the opposite compartment for 25 min. Rats in the saline group were injected with cocaine during both odd and even days. On the test day (day 9), rats from the saline and cocaine conditioned groups were injected with saline and were allowed to explore the entire apparatus for 10 min. Time spent in each compartment was recorded and evaluated. On successful expression of a place preference, rats were randomly assigned to an abstinence group [short abstinence (SA): 8 d after the initial CPP test or prolonged abstinence (PA): at least 30 d after the initial CPP test] and were tested for CPP (+) or remained in their home cages (-) for 24 h, after which time, the brains were dissected and mPFC slices were prepared for patch-clamp electrophysiology experiments (Fig. 1*A*). Saline rats (Ctrl) remained in their home cages for 8 d (SA) following the initial preference test and were allowed to explore the CPP apparatus 10 min before tissue processing for electrophysiology experiments (Fig. 1*A*).

### Brain slice preparation and electrophysiology

Saline or cocaine-treated rats were deeply anesthetized with isoflurane, brains were removed, and coronal PFC slices (300 μm) were cut on a vibratome (Leica, VT1200S) in ice-cold sucrose-containing ACSF: 200 mM sucrose, 1.9 mM KCl, 1.2 mM Na_2_HPO_4_, 33 mM NaHCO_3_, 6 mM MgCl_2_, 0.5 mM CaCl_2_, 10 mM D-glucose, and 0.4 mM ascorbic acid. Slices were incubated at 32°C for at least 1 h in a solution consisting of 120 mM NaCl, 2.5 mM KCl, 1.25 mM NaH_2_PO_4_, 25 mM NaHCO_3_, 4 mM MgCl_2_, 1 mM CaCl_2_, 10 mM D-glucose, and 0.4 mM ascorbic acid. Then the slices were transferred to a recording chamber. Recordings were performed at room temperature using a recording ACSF consisting of 126 mM NaCl, 2.5 mM KCl, 1.4 mM NaH_2_PO_4_, 25 mM NaHCO_3_, 2.0 mM CaCl_2_, 1.3 mM MgCl_2_, 10 mM D-glucose, and 0.4 mM ascorbic acid at a rate of 2-3 ml/min. All ACSF solutions were constantly aerated with a mixture of 95% O_2_–5% CO_2_ (pH 7.2, 300-310 mOsm).

Whole-cell voltage-clamp recordings were obtained from visually identified pyramidal neurons in layers 5/6 of the PL-mPFC and IL-mPFC using differential interference contrast optics (Axioskop 2, Zeiss) attached to a camera (Dage-MTI). Recordings electrodes (2.5-3 MOhm pipette resistance) were filled with 130 mM CsCl, 10 mM HEPES, 2 mM MgCl_2_, 0.5 mM EGTA, 2 mM Na_2_ATP, 0.3 mM Na-GTP, 2 mM QX-314, 10 mM phosphocreatine, and 0.1 mM spermine; 290 mOsmol.

### Data collection and analysis

Time spent in each compartment was recorded with Med Associates software on the preference (CPP), SA, and PA tests. Data were converted into a preference ratio to definitively assert a compartment preference. The ratio was calculated with the following formula: [(time in CS+/(time in CS- + CS+)) × 100] (Reichel and Bevins, 2008). Saline control rats values were randomly assigned a CS+ and CS- compartment for comparison purposes (Reichel et al., 2010). Preference ratios were then compared against a hypothetical mean of 50%. A preference score of 50% indicated a lack of compartment preference; a value >50% indicated a preference for the drug paired compartment. Rats that failed to show a preference ratio >50% were excluded from the study (*n* = 8).

Electrophysiological recordings were obtained with a Multiclamp 700B amplifier (Molecular Devices). Signals were low-pass filtered at 3 kHz and digitized at 10 kHz. Data were stored on PC for off-line analysis. Data acquisition was performed using Axograph-X software (J. Clements). Analysis of spontaneous EPSCs (sEPSCs) and evoked EPSCs (eEPSC) peak amplitude data were done in Mini Analysis (v6.0.7; Synaptosoft). Each parameter was measured in the following order: (1) sEPSCs and (2) AMPA-eEPSC/rectification index (RI). (1) Briefly, membrane potential was held at −70 mV, and glutamate-mediated events were pharmacologically isolated by adding picrotoxin (50 μM) to the bath. Series resistance (Rs) was continuously monitored by applying a small hyperpolarizing voltage step (-5 mV, 50 ms), and recordings that exceeded Rs > 30 MΩ were discarded. sEPSC recordings consisted of five sweeps/10 s-long recordings that were analyzed for amplitude and frequency of detected events. For sEPSC kinetics, all detected events per cell were used to obtain average rise (ms), decay (ms), and area (pA/ms) for all experimental groups. (2) The membrane potential was slowly shifted to +40 mV and 50 µM D-APV (NMDA receptor blocker) was added for at least 5 min to isolate AMPA-mediated responses. Eight to ten isolated AMPA responses were recorded at +40 mV and the membrane potential was slowly shifted to -70 mV, where 8-10 responses were recorded. RI was calculated as the average eEPSC at -70 mV over the average eEPSC at +40 mV.

Our a priori research questions were: (1) whether the treatment groups were different from the control group (Ctrl vs SA and Ctrl vs PA); (2) whether the treatment groups were different from each other (SA vs PA); and (3) do differences exist between the treatment groups if rats were tested (reexposed to the conditioning context) or not [SA (-) vs SA (+) and PA (-) vs PA (+)]. For statistical comparisons, multiple *t* tests with corrections (Bonferroni-Dunn method) for multiple comparisons were used to compare average saline control electrophysiological measures from PL- or IL-mPFC neuron recordings versus cocaine-treated groups. Two-tailed, unpaired *t* tests were performed to compare sEPSC amplitude and frequency values of saline control PL- versus IL–PFC pyramidal neurons. Individual two-sample Kolmogorov-Smirnov tests were used to detect shifts between cocaine-treated groups and saline control sEPSC amplitude and interevent interval distributions. Differences of α ≤ 0.05 were considered statistically significant. All data are presented as mean ± SEM.

### Drugs

Cocaine HCl was gifted from the National Institutes of Health and dissolved in 0.9% saline. QX 314 chloride was purchased from Tocris. Spermine and picrotoxin were purchased from Sigma-Aldrich. D-APV was purchased from Abcam. All drugs used in the electrophysiological recordings were dissolved in recording ACSF and bath applied except for QX 314 and spermine, which were dissolved into internal ACSF and stocks were further diluted in the internal solution the day of recording.

## Results

We used the cocaine-CPP paradigm to assess the cocaine-associated contextual cue effects on glutamatergic synaptic transmission in mPFC. Whole-cell voltage clamp recordings were performed from layer 5/6 pyramidal neurons of PL- or IL-mPFC in brain slices of adult male rats (Fig. 1*A*,*B*).

### Behavior: cocaine-induced CPP

During the PC test, rats spent similar amounts of time in the black and white compartments confirming the unbiased construction of the testing apparatus (Fig. 1*C*). Saline control rats did not show a conditioned response, given that preference ratios did not differ from chance performance on the initial preference test (*t*_(15)_ = 1.103, *p* > 0.05) or the SA test (*t*_(11)_ = 0.71, *p* > 0.05; Fig. 1*D*). Rats conditioned with 20 mg/kg i.p. cocaine displayed a preference for the cocaine paired compartment as indicated by a preference ratio greater than chance performance on the initial test for place conditioning (*t*_(38)_ = 4.194, *p* = 0.0002) the SA test [SA(+), *t*_(32)_ = 4.835, *p* < 0.0001] and the PA test [PA(+), *t*_(12)_ = 2.255, *p* = 0.0436; Fig. 1*D*]. In summary, these results suggest that in the course of abstinence, when cocaine-treated rats are reexposed to the cocaine-associated context under drug free conditions, the difference in preference ratios from the respective saline control group becomes higher with longer abstinence. These results could be interpreted as a disruption of the rewarding memory of the initial cocaine experience, leading to a slight decrease in cocaine seeking behavior.

### Electrophysiology: sEPSCs

We measured frequency and amplitude of sEPSCs in our experimental and control groups to investigate the basal differences between PL- and IL-mPFC deep layer 5/6 pyramidal neurons and to assess the changes in synaptic glutamate transmission after two different abstinence time points (SA, 8 d of abstinence; and PA, 30 d of abstinence) from cocaine-induced CPP.

**Figure 2.**
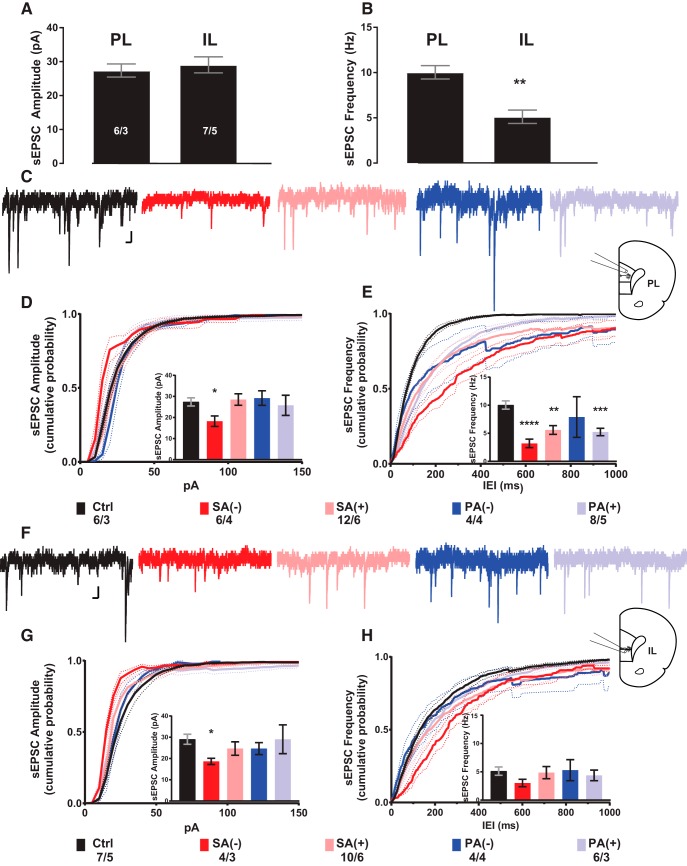
Cocaine-associated context experience-dependent alterations in sEPSC properties in deep layer 5/6 pyramidal neurons of PL -and IL-mPFC. ***A***, ***B***, Comparison of sEPSC amplitude and frequency (mean ± SEM pA and Hz, respectively) from PL-mPFC versus IL-mPFC deep layer 5/6 pyramidal neurons in saline-treated control rats. ***C***, Representative traces of sEPSC recordings from PL-mPFC pyramidal neurons for each experimental group with a diagram for recording location on brain slice. ***D***, ***E***, Comparison of sEPSC amplitude and frequency cumulative probability distributions from PL-mPFC pyramidal neurons for each experimental group. Insets show average data for sEPSC amplitude and frequency (mean ± SEM pA and Hz, respectively). ***F***, Representative traces of sEPSC recordings from IL-mPFC pyramidal neurons for each experimental group with diagram for recording location on brain slice. ***G***, ***H***, Comparison of sEPSC amplitude and frequency cumulative probability distributions from PL-mPFC pyramidal neurons for each experimental group. Insets show average data for sEPSC amplitude and frequency (mean ± SEM pA and Hz, respectively). Statistics on bar graphs represent adjusted *p* values calculated from multiple *t* tests against saline control measurements corrected for multiple comparisons (Bonferroni-Dunn method). Cumulative probability distributions were tested individually against saline control distributions for each sEPSC measurement; *p < 0.05, **p < 0.005, ***p < 0.001, ****p < 0.0005. Numbers represent cells/rats. Scale bars: 100 ms (horizontal), 10 pA (vertical).

When comparing amplitude and frequency of sEPSCs between PL- and IL-mPFC in saline animals, we found no statistical differences in the amplitude of spontaneous events but we found that IL-mPFC neurons exhibit a significantly lower frequency of sEPSCs than the PL-mPFC (Ctrl: *t*_(11)_ = 4.676, *p* = 0.0007; Fig. 2*A*,*B*).

### Cocaine-induced CPP after SA or PA: sEPSCs amplitude

In the PL-mPFC, we found a significant reduction in the amplitude of sEPSCs relative to Ctrl levels exclusively in neurons from SA(-) rats that were not reexposed to the cocaine-associated context [SA(-): *t*_(10)_ = 2.914, *p* = 0.031; Fig. 2*D*, inset]. Similarly, in the IL-mPFC, we observed a reduction in the amplitude of sEPSCs only in SA(-) rats (*t*_(9)_ = 3.086, *p* = 0.026; Fig. 2*G*, inset).

### Cocaine-induced CPP after SA or PA: sEPSCs frequency

Our results showed that PL-mPFC deep layer pyramidal neurons exhibit a significant reduction in the frequency of sEPSCs in all cocaine-CPP groups relative to saline values, except for the PA(-) group: [SA(-): *t*_(10)_ = 6.498, *p* = 0.0001; (SA+): *t*_(16)_ = 3.618, *p* = 0.0046; PA(+): *t*_(12)_ = 4.815. *p* = 0.0008; Fig. 2*E*, inset]. In contrast to the findings in the PL-mPFC, cocaine-induced CPP followed by abstinence did not elicit significant changes in the frequency of sEPSCs in IL-mPFC neurons (Fig. 2*H*, inset).

Our findings suggest that under basal conditions, pyramidal neurons located in deep layers of the PL-mPFC receive higher levels of glutamate input compared to IL-mPFC neurons. Moreover, cocaine-CPP followed by abstinence elicits different cortical adaptations in glutamate synaptic transmission in mPFC neurons after short and long abstinence periods. SA elicited decreases in the amplitude of glutamate-mediated currents, suggesting a postsynaptic decrease in receptor levels in PL- and IL-mPFC neurons in rats that were not reexposed to the cocaine-associated context (were not tested for CPP). In contrast, we found a decrease in the frequency of excitatory synaptic inputs only in PL-mPFC neurons. This decrease was independent of reexposure or deprivation of the cocaine-associated context and suggests a maintained pharmacological mechanism perhaps originating from the repeated cocaine exposure.

### sEPSC kinetics

**Figure 3.**
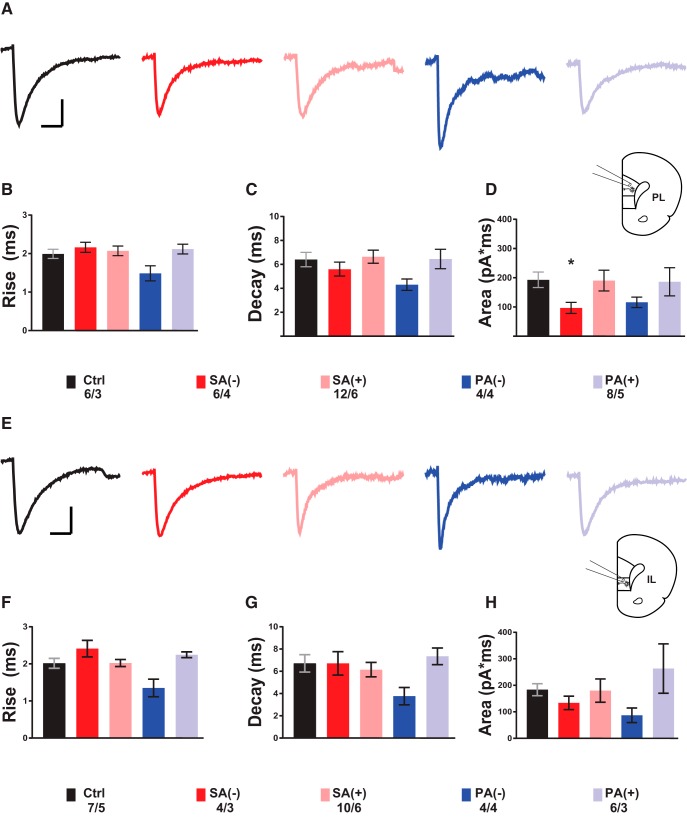
Cocaine-associated context experience-dependent alterations in the kinetics of sEPSCs in deep layer 5/6 pyramidal neurons of PL- and IL-mPFC. ***A***, Average traces from single events in representative sEPSC recordings from PL-mPFC pyramidal neurons for each experimental group with diagram for recording location on brain slice. ***B***, ***F***, Average rise time from all events detected in sEPSC recordings for PL- and IL-mPFC pyramidal neurons respectively (mean ± SEM ms). ***C***, ***G***, Average decay time from all events detected in sEPSC recordings for PL- and IL-mPFC pyramidal neurons respectively (mean ± SEM ms). ***D***, ***H***, Average area under the curve from all events detected in sEPSC recordings for PL- and IL-mPFC pyramidal neurons respectively (mean ± SEM pA/ms). ***E***, Average traces from single events in representative sEPSC recordings from PL-mPFC pyramidal neurons for each experimental group with diagram for recording location on brain slice. Statistics on bar graphs represent adjusted *p* values calculated from multiple *t* tests against saline control measurements corrected for multiple comparisons (Bonferroni-Dunn method); *p* < 0.05. Numbers represent cells/rats. Scale bars: 10 ms (horizontal), 10 pA (vertical).

To further address cocaine-CPP changes in spontaneous glutamate transmission, we used the average sEPSC event kinetics as an indirect measure of glutamate receptor dynamics (Hollmann et al., 1989; Keller et al., 1991; Tomita, 2010; Traynelis et al., 2010). Analysis of individual events from sEPSC recordings in PL-mPFC layer 5/6 pyramidal neurons revealed a significant decrease in the area under the curve in sEPSC events from SA(-) PL-mPFC neurons (*t*_(10)_ = 2.942, *p* = 0.029) Fig. 3*D*. Consistent with the difference between mPFC subregions, in the IL-mPFC we found no difference in the rise time and decay time for the average of all detected sEPSC events, Fig. 3.

In summary, our results showed that the activation and inactivation rate of sEPSCs are, for the most part, unaltered in PL- and IL-mPFC neurons of saline and cocaine-treated rats. The significant decrease in area under the curve observed in 8-d abstinent rats that were not reexposed to the cocaine-conditioned context [SA(-)] follows the decrease in sEPSC amplitude and is similarly reversed on context reexposure. Since changes in sEPSC area under the curve are thought to represent the net charge transfer during an ionotropic glutamate receptor-mediated event, this result suggests that SA after cocaine conditioning decreases the glutamatergic excitatory strength in PL-mPFC deep layer 5/6 pyramidal neurons (Keller et al., 1991).

### RI

**Figure 4.**
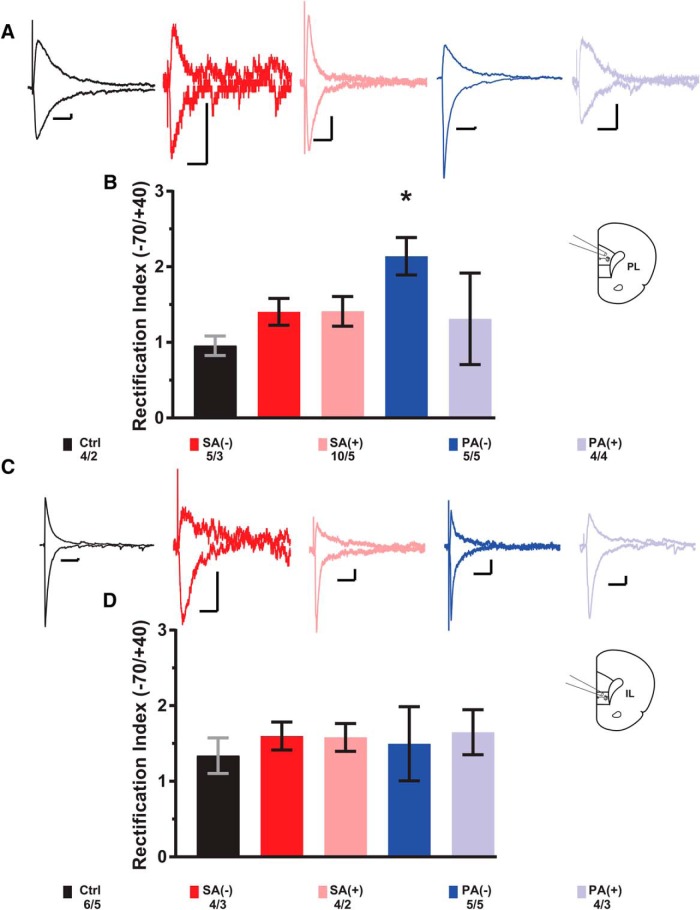
Cocaine-associated context experience-dependent alterations in the RI in deep layer 5/6 pyramidal neurons of PL and IL-mPFC. ***A***, ***B***, Representative traces from single evoked EPSC recordings from PL- and IL-mPFC pyramidal neurons, respectively, at +40 mV (outward) and -70 mV (inward) for each experimental group with diagram for recording location on brain slice. ***B***, ***D***, Average RI values calculated as evoked EPSC amplitude at -70 mV over +40 mV membrane potentials for PL- and IL-mPFC pyramidal neurons respectively. Statistics on bar graphs represent adjusted *p* values calculated from multiple *t* tests against saline control measurements corrected for multiple comparisons (Bonferroni-Dunn method); *p* < 0.05. Numbers represent cells/rats. Scale bars: 100 ms (horizontal), 10 pA (vertical).

Previous studies have shown that protracted abstinence increases Ca^2+^-permeable AMPARs (CP-ARs) in the NAc of cocaine self-administering rats (Conrad et al., 2008; McCutcheon et al., 2011) and alterations in AMPA receptor Ca^2+^ permeability has been shown to increase neuronal excitability (Li et al., 2012). To explore if similar neuroadaptations occur in PL- and/or IL-mPFC, we assessed the RI in our cocaine-CPP rats as an indirect measure of CP-AR levels, where an increase in RI suggests a higher contribution of CP-ARs and a decrease in RI suggests less contribution of CP-ARs. Our results show a significant increase in the RI in PL-mPFC from PA(-) neurons compared to saline control values (*t*_(7)_ = 0.0117, *p* = 0.0207; Fig. 4*B*). This data suggests that only protracted abstinence (30 d after the initial CPP test) from cocaine treatment produces an increase in RI, similar to what has been previously reported in NAc medium spiny neurons (MSNs) from long-access cocaine self-administering rats. When RI was assessed in IL-mPFC neurons, there were no significant differences from saline controls in any of the cocaine-treated groups (Fig. 4*D*).

In summary, our results showed for the first time that layer 5/6 pyramidal neurons from the PL- and IL-mPFC are intrinsically different in their excitatory synaptic activity (see differences in basal sEPSC frequency). Furthermore, we showed that cocaine-CPP induces differential effects between PL-mPFC and IL-mPFC neurons that are dependent on the length of abstinence. Experiments showed general mPFC decreases in excitatory synaptic inputs to pyramidal cells following SA (8 d after the initial CPP test) and changes in glutamate receptor dynamics after PA (30 d after the initial CPP test). Moreover, after a period of PA, we found a PL-specific increase in the contribution of CP-ARs in response to electrical stimulation of glutamate terminals innervating layer 5/6 pyramidal neurons.

## Discussion

Our results are the first to show that deep layer pyramidal neurons from PL- and IL-mPFC differ in the frequency of their excitatory synaptic inputs, with the PL-mPFC exhibiting higher frequency of excitatory inputs than the IL-mPFC. Furthermore, our results showed that cocaine conditioning followed by abstinence elicits differential effects on PL- and IL-mPFC pyramidal neurons and that these glutamatergic changes could represent synaptic alterations that mediate the retention of the cocaine-associated rewarding memory.

### Presynaptic markers

It has been proposed that changes in frequency of sEPSCs reflect modifications at the presynaptic level (Engelman and MacDermott, 2004; Costa et al., 2017). Interestingly, our results showed that PL-mPFC neurons exhibit higher basal frequency of sEPSCs compared to IL-mPFC neurons. This reduced basal frequency of synaptic glutamate currents in IL-mPFC neurons may be a phenotype of this distinct neuronal population arising from differences in dendritic cytoarchitecture and glutamate afferents innervating this region (Van Eden and Uylings, 1985; Hoover and Vertes, 2007). In addition, the reduced basal frequency of sEPSCs in IL-mPFC neurons could preclude any further reduction in this neuronal population, thus supporting the lack of effects observed following cocaine experience, abstinence and cocaine-context reexposure. It is tempting to speculate that higher excitatory drive onto PL-mPFC neurons plays a role in the retention of the rewarding effects of cocaine after abstinence.

We show that cocaine-induced CPP elicits a significant reduction in the frequency of sEPSCs in PL-mPFC in all groups except the PA(-) group. In contrast, we did not find changes in sEPSC frequency in IL-mPFC neurons. Based on these results, we propose that abstinence from cocaine experience selectively alters excitatory inputs to the PL-mPFC. A recent study showed that cocaine-CPP produced an increase in sEPSC frequency in PL-mPFC neurons (Otis and Mueller, 2017). The discrepancy with our results can be explained by the length between the last conditioning session and the time of recording; their recordings were performed 4 d after the last conditioning session and we recorded from rats after at least 8 d of abstinence. Work from Hoover and Vertes (2007) show distinct patterns of excitatory afferents from multiple limbic, thalamic and cortical nuclei innervating PL- and IL-mPFC neurons, and thus future studies should selectively target each excitatory input to investigate which inputs are relevant to the induction of these specific changes in glutamate transmission after abstinence from cocaine-CPP (Hoover and Vertes, 2007) additional experiments should focus on elucidating the origin of our reported synaptic changes by implementing a combination of electrophysiological assays that target presynaptic analysis with postsynaptic measures(Graziane and Dong, 2016).

### Postsynaptic markers

Changes in amplitude of sEPSCs are generally associated with differences in the levels of postsynaptic receptors and/or changes in the dynamics of the receptors (Engelman and MacDermott, 2004; Costa et al., 2017). A comparison of the average sEPSC amplitude found that cocaine-CPP elicited only a reduction in the amplitude of sEPSCs in PL- and IL-mPFC pyramidal cells after SA without reexposure to the cocaine-associated context [SA(-)], but this decrease was not maintained long-term. A recent study (Otis and Mueller, 2017) showed an increase in sEPSC amplitude in PL-mPFC neurons following cocaine-CPP. The difference with our results could be explained by a memory reconsolidation mechanism that was not activated in our study since our rats remained in the home cage before the recordings. The presence of the reduction in amplitude of sEPSCs in both mPFC subregions suggests a common short-term neuroadaptation by which cocaine experience, in combination with deprivation from the cocaine-associated context, decreases the levels of postsynaptic glutamate receptors. After SA, retrieval of the cocaine-context association memory could be enough to disrupt this adaptation. Moreover, because our rats were tested at multiple intervals for the retention of cocaine-CPP, it is possible that at longer periods of abstinence, this modification is either not necessary for the expression of cocaine-induced CPP or has been disrupted by the emergence of an extinction memory.

Changes in the kinetics of sEPSC events can be indicative of alterations in receptor subunit composition and their interactions with auxiliary subunits as well as alternative RNA splicing and post-translational modifications (Dingledine et al., 1999; Tomita, 2010; Granger et al., 2011; Stincic and Frerking, 2015). Using the CPP protocol with cocaine exposure followed by different periods of abstinence, we found that only PL-mPFC neurons exhibited a decrease in the area under the curve in the SA (-) group. Given the low contribution of NMDA receptors at -70 mV, we interpreted these changes in sEPSC area as alterations in AMPA glutamate receptor dynamics and a putative by-product of the combination of the factors mentioned above. Moreover, these changes appear to be dependent on SA and deprivation from the cocaine-conditioned context.

It is important to state that a portion of our experimental groups have small samples sizes; therefore, in future studies, we will attempt to expand these experiments to parse out any underpowered effects that might have gone undetected.

### RI

Previous reports have shown that long-access cocaine SA followed by PA elicits an increase in the number of calcium permeable AMPA receptors (CP-ARs) in NAc MSNs (Conrad et al., 2008), thus providing a synaptic marker of the incubation of cocaine craving in this brain region. Based on these studies and the influence that PFC projection neurons have within the drug-seeking circuit, we assessed changes in RI (as a relative measure of CP-AR’s contribution) in our different experimental groups. We found that cocaine-CPP followed by abstinence elicited a significant increase in RI only in the PL-mPFC [PA(-)]. This result suggests that longer periods of withdrawal from the cocaine experience are required to produce an increase in RI, similar to what has been previously reported in NAc MSNs from long-access cocaine self-administering rats (Conrad et al., 2008). An increase in surface level expression of CP-ARs, with enhanced single channel conductance, could increase the excitability of mPFC pyramidal neurons in similar fashion to the effects of GluR2-lacking AMPARs reported in PVN neurons in spontaneous hypertensive rats (Li et al., 2012). Our results suggest that PA alters the activity of PL-mPFC neurons via an unknown mechanism leading to a compensatory increase in AMPA receptor RI that can potentially drive the retention of cocaine-induced CPP.

Silent synapses, synapses devoid of AMPA receptors, were initially reported in hippocampal CA1 neurons in studies demonstrating that their “unsilencing” required the insertion of AMPA receptors on induction of LTP (Isaac et al., 1995; Liao et al., 1995). Huang and colleagues established an association between silent synapses and *in vivo* cocaine experience, where the salience attributed to the drug experience is sufficient to generate *de novo* silent synapses (Huang et al., 2009). Cocaine-induced silent synapse formation has been shown to generate a permissive state for remodeling of the NAc neurocircuits in several cocaine-related behaviors including CPP, locomotor sensitization and cue-induced reinstatement of cocaine self-administration (Brown et al., 2011; Lee et al., 2013; Ma et al., 2014; Dong, 2015; Shukla et al., 2017). Particularly relevant to our study is the evidence of silent synapse-based circuit remodeling in the mPFC-NAc pathway during cocaine craving, showing that maturation of the IL-mPFC-NAc pathway requires the recruitment of CP-AMPARs and that maturation of the PL-mPFC-NAc pathway requires insertion of non-CP-AMPARs. Reversing excitatory synapse remodeling with optogenetic stimulation of the IL-mPFC to NAc shell and the PL-mPFC to NAc core pathways can potentiate or inhibit incubation of cocaine craving, respectively (Ma et al., 2014). It remains to be studied whether similar forms of circuit remodeling occur at the level of mPFC afferents. The following questions remains to be answered: do cocaine-context associations require the generation of *de novo* silent synapses in deep layer mPFC pyramidal neurons, and does this PL- vs IL-mPFC dichotomy prevail upstream from the NAc?

## Conclusion

**Figure 5.**
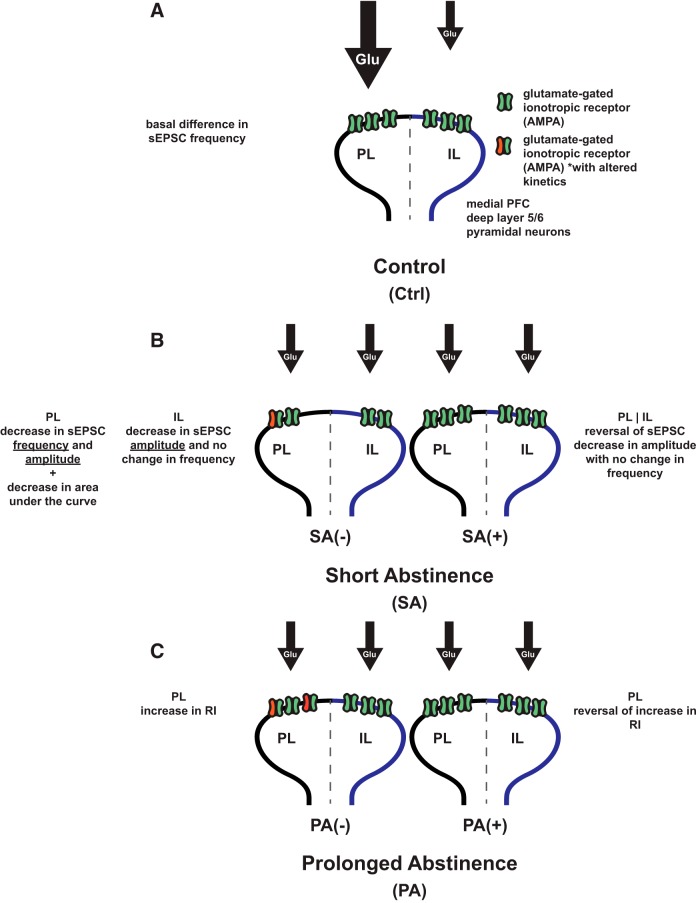
Summary diagram. Targeted effects of cocaine-CPP on PL- versus IL-mPFC deep layer 5/6 pyramidal neurons after SA or PA. ***A***, Basal PL/IL difference in sEPSC frequency in cocaine naïve control rats: higher frequency of sEPSC in PL- neurons suggest more glutamate inputs to PL- versus IL-mPFC neurons, while no difference in the amplitude of sEPSC suggest no difference in postsynaptic receptor levels. ***B***, SA from cocaine-CPP (left); SA(-): decreases sEPSC amplitude in PL- and IL –mPFC neurons implying a decrease in postsynaptic receptor levels. SA also produces a decrease in sEPSC amplitude and a decrease in the area under the curve of the average sEPSC. Reexposure to the conditioning context after SA (right); SA(+): reversal of changes to PL- and IL-mPFC neurons sEPSC amplitude to control levels. ***C***, PA from cocaine-CPP(left); PA(-): PL- neurons show an increase in AMPA receptor RI suggesting higher levels of GluA2-lacking AMPARs. Reexposure to the conditioning context after PA(right); PA(+): changes in average sEPSC kinetics are reversed in PL- and IL-mPFC neurons. Increase in AMPAR RI in PL-mPFC neurons is reversed to control levels.

PL- and IL-mPFC deep layer 5/6 pyramidal neurons differ in their excitatory inputs, with PL-mPFC exhibiting higher basal frequency of sEPSCs, which suggests an important role of this subcortical region for the neuroplasticity of addiction. Cocaine-CPP elicits different neuroadaptations in mPFC neurons depending on the length of abstinence, and the specific changes are detailed in the summary diagram (Fig. 5). General adaptations appeared after SA (8 d after the initial CPP test) in both PL- and IL-mPFC neurons, suggesting the maintenance of the pharmacological effects of cocaine, whereas alterations in frequency of glutamate inputs after SA were specific to PL-mPFC neurons. In both cases, these effects were only present in rats deprived from context reexposure and were not present after PA (30 d after the initial CPP test). PA produces PL-specific changes in CP-AMPARs, suggesting a time sensitivity to the effects of cocaine-induced CPP in mPFC synaptic glutamate transmission.

## Synthesis

Reviewing Editor: Karen Szumlinski, University of California at Santa Barbara Decisions are customarily a result of the Reviewing Editor and the peer reviewers coming together and discussing their recommendations until a consensus is reached. When revisions are invited, a fact-based synthesis statement explaining their decision and outlining what is needed to prepare a revision will be listed below. The following reviewer(s) agreed to reveal their identity: Yan Dong, Wen-Jun Gao

Following consultation with 2 reviewers with expertise in neuronal physiology and the neurobiology of cocaine addiction, I have reached the decision of Major Revision. This decision reflects our opinion that the results of your study provide new insight into the cellular changes within mPFC in regulating cocaine reward, although Reviewer 2 did contend that the study was not highly novel, given prior work in the field implicating altered AMPA function, in particular, CP-AMPA function, in cocaine addiction-related behaviors. However, we all agree that relative to the NAC, there is relatively little work on the physiological and molecular changes that occur within the mPFC proper and thus, your study begins to fill this knowledge gap. The Reviewers and I agree that the statistical approaches employed are not appropriate and the data should be re-analyzed using ANOVAs, when appropriate. The likelihood of detecting significant effects and interactions will be greater if the sample sizes employed in the analyses were increased, as suggested by Reviewer 1. In the very least, the small sample sizes should be discussed as a limitation of the study. Reviewer 2 argued for greater exploration of the observation that both sEPSC frequency and amplitude were changed. Specifically, this reviewer considered an examination of mEPSC frequency as particularly important based on the literature positing that CP-AMPA changes are likely post-synaptic.

Additional Specific Comments:

Reviewer 1:

1) Some important experimental information, e.g., at which drug abstinence time points the cellular measurements were taken. It was clear in figures, but not always clear in the text.

2) Fig. 3D: it was stated in the text p=0.065, but in the figure a “*” is labeled to show significance. The bar graph looks like it is significance. Did I miss anything? Nonetheless, does this data set suggest that there is a potential effect of saline (saline+ vs. saline-)?

3) The manuscript deserves a careful proofread.

Reviewer 2:

Major comments

1) The changes in both frequency and amplitude of the sEPSC suggest an involvement of both presynaptic and postsynaptic mechanisms. However, the authors did not explore this possibility further. At least, to determine post-synaptic effects, changes in miniature EPSC should be recorded.

2. The quality of the recordings, especially the data showed Figure 4, was not good. The results were not very convincing.

Minor issues:

1. The results showed in Figure 2C were hard to be distinguished.

2. P18 Lines 478 and 480: both layer Ⅵ/Ⅶ and layer5/6 were used in the text. Be consistent.
